# Supplementary material for: A Rapid and Specific Genotyping Platform for Plasmodium falciparum Chloroquine Resistance via Allele-Specific PCR with a Lateral Flow Assay
Source: Microbiol Spectr. 2022 Apr 13;10(2):e02719-21. doi: 10.1128/spectrum.02719-21 (PMC9045167; doi:10.1128/spectrum.02719-21)
Supplement: SUPPLEMENTAL FILE 1 — Fig. S1 and Fig. S2. Download spectrum.02719-21-s001.pdf, PDF file, 0.6 MB [file spectrum.02719-21-s001.pdf]

Supporting information

Fig. S1 Recombinant plasmid construction and identification. A: Double digestion with the restriction endonucleases *BamHI* and *XhoI*. Marker represents the DL5000 DNA ladder (100 bp, 250 bp, 500 bp, 750 bp, 1 kb, 2 kb, 3 kb, 4 kb and 5 kb); pDNA represents plasmid DNA. 1 and 2 represent the recombinant plasmids *pUC57*-Pfcrt-CVMNK (wild-type) and *pUC57*-Pfcrt-CV**IET** (mutant), respectively; **B**: DNA sequencing.

Fig. S2 Analytical sensitivity for multiple infections. The initial concentration of wild-type and mutant-type plasmids was 3.38×10^7 copies/μl; The different ratios of 1:9, 2:8, 3:7, 4:6, 5:5, 6:4, 7:3, 8:2 and 9:1 indicate different combinations of wild-type and mutant-type, respectively; W represents wild-type primer, M represents mutant-type primer; Marker indicates the DNA molecular marker, including 100 bp, 300 bp, 500 bp, 700 bp, 900 bp and 1200 bp. The brightest band was 700 bp with a mass concentration of 83 ng and the rest of the bands were 42 ng; C and T represent the control line and test line respectively.

Table S1 Comparison between nested PCR with sequencing and AS-PCR-LFA for genotype detection in the *pfcrt* gene with clinical isolates of *Plasmodium falciparum*.

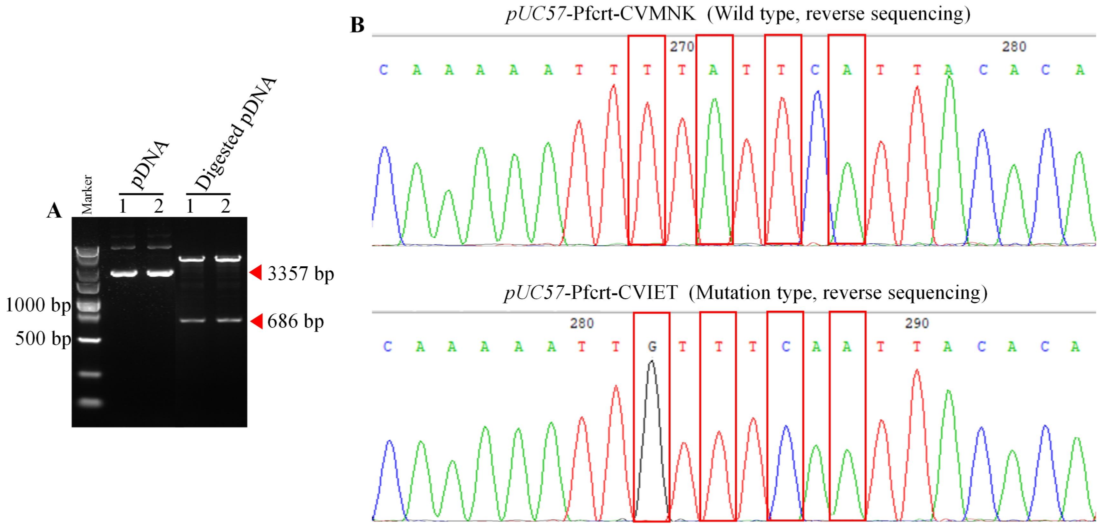

Fig. S1 Recombinant plasmid construction and identification. A: Double digestion with the restriction endonucleases *BamHI* and *XhoI*. Marker represents the DL5000 DNA ladder (100 bp, 250 bp, 500 bp, 750 bp, 1 kb, 2 kb, 3 kb, 4 kb and 5 kb); pDNA represents plasmid DNA. 1 and 2 represent the recombinant plasmids *pUC57*-Pfcrt-CVMNK (wild-type) and *pUC57*-Pfcrt-CV**IET** (mutant), respectively; **B**: DNA sequencing.

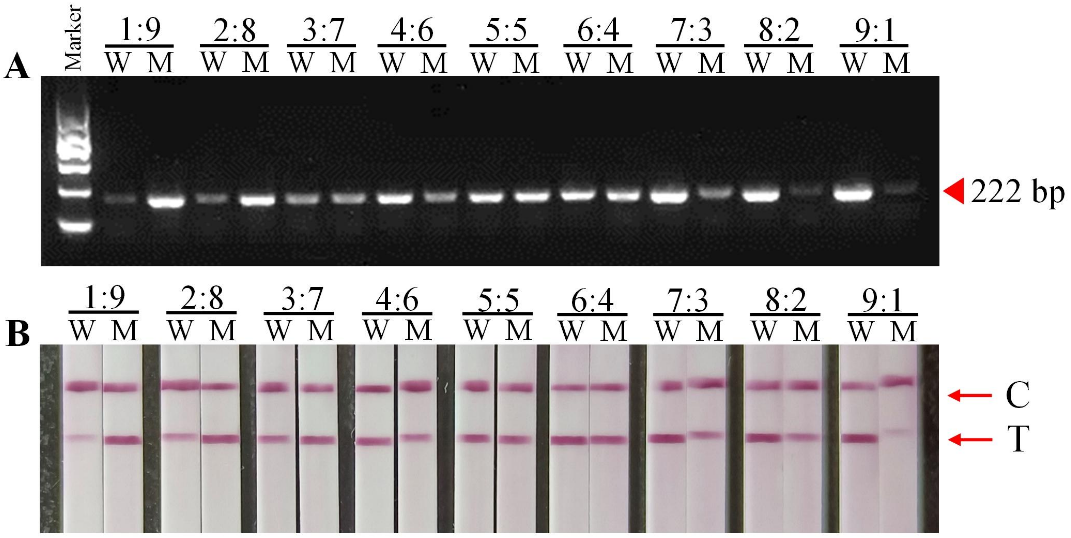

Fig. S2 Analytical sensitivity for multiple infections. The initial concentration of wild-type and mutant-type plasmids was 3.38×10^7 copies/μl; The different ratios of 1:9, 2:8, 3:7, 4:6, 5:5, 6:4, 7:3, 8:2 and 9:1 indicate different combinations of wild-type and mutant-type, respectively; W represents wild-type primer, M represents mutant-type primer; Marker indicates the DNA molecular marker, including 100 bp, 300 bp, 500 bp, 700 bp, 900 bp and 1200 bp. The brightest band was 700 bp with a mass concentration of 83 ng and the rest of the bands were 42 ng; C and T represent the control line and test line respectively.
